# Disparities in Access to Liver Transplant Referral and Evaluation among Patients with Hepatocellular Carcinoma in Georgia

**DOI:** 10.1158/2767-9764.CRC-23-0541

**Published:** 2024-04-22

**Authors:** Katherine Ross-Driscoll, Arrey-Takor Ayuk-Arrey, Raymond Lynch, Lauren E. McCullough, Giorgio Roccaro, Lauren Nephew, Jonathan Hundley, Raymond A. Rubin, Rachel Patzer

**Affiliations:** 1 Division of Transplantation, Department of Surgery, Indiana University School of Medicine, Indianapolis, Indiana.; 2 Center for Health Services Research, Regenstrief Institute, Indianapolis, Indiana.; 3 Department of Epidemiology, Rollins School of Public Health, Atlanta, Georgia.; 4 Division of Transplantation, Department of Surgery, Pennsylvania State University School of Medicine, Hershey, Pennsylvania.; 5 Winship Cancer Institute, Emory University, Atlanta, Georgia.; 6 Division of Digestive Diseases, Department of Medicine, Emory University School of Medicine, Atlanta, Georgia.; 7 Division of Gastroenterology and Hepatology, Department of Medicine, Indiana University, Indianapolis, Indiana.; 8 Piedmont Transplant Institute, Piedmont Healthcare, Atlanta, Georgia.; 9 Regenstrief Institute, Indianapolis, Indiana.

## Abstract

Liver transplantation offers the best survival for patients with early-stage hepatocellular carcinoma (HCC). Prior studies have demonstrated disparities in transplant access; none have examined the early steps of the transplant process. We identified determinants of access to transplant referral and evaluation among patients with HCC with a single tumor either within Milan or meeting downstaging criteria in Georgia.

Population-based cancer registry data from 2010 to 2019 were linked to liver transplant centers in Georgia. Primary cohort: adult patients with HCC with a single tumor ≤8 cm in diameter, no extrahepatic involvement, and no vascular involvement. Secondary cohort: primary cohort plus patients with multiple tumors confined to one lobe. We estimated time to transplant referral, evaluation initiation, and evaluation completion, accounting for the competing risk of death. In sensitivity analyses, we also accounted for non-transplant cancer treatment.

Among 1,379 patients with early-stage HCC in Georgia, 26% were referred to liver transplant. Private insurance and younger age were associated with increased likelihood of referral, while requiring downstaging was associated with lower likelihood of referral. Patients living in census tracts with ≥20% of residents in poverty were less likely to initiate evaluation among those referred [cause-specific hazard ratio (csHR): 0.62, 95% confidence interval (CI): 0.42–0.94]. Medicaid patients were less likely to complete the evaluation once initiated (csHR: 0.53, 95% CI: 0.32–0.89).

Different sociodemographic factors were associated with each stage of the transplant process among patients with early-stage HCC in Georgia, emphasizing unique barriers to access and the need for targeted interventions at each step.

**Significance:**

Among patients with early-stage HCC in Georgia, age and insurance type were associated with referral to liver transplant, race, and poverty with evaluation initiation, and insurance type with evaluation completion. Opportunities to improve transplant access include informing referring providers about insurance requirements, addressing barriers to evaluation initiation, and streamlining the evaluation process.

## Introduction

Hepatocellular carcinoma (HCC) is a major cause of cancer death in the United States (1). According to the American Association for the Study of Liver Diseases practice guidelines, liver transplantation is an effective therapy for patients with HCC meeting the Milan criteria (single tumor ≤5 cm or no more than three tumors each ≤3 cm, no vascular invasion, and no extrahepatic involvement; refs. 2, 3). In some cases, patients with tumors outside these criteria can receive therapies to reduce tumor burden and achieve transplant eligibility, also called downstaging (3). Prompt access to liver transplantation is of particular importance for patients with HCC, as patients can become ineligible for transplant if their disease progresses.

Receiving a transplant is a multistep process requiring referral to a transplant center, completion of a medical and psychosocial evaluation, and selection for placement on the deceased donor waiting list (listing) or identification of an appropriate living donor. Prior studies have demonstrated substantial racial (4–7), socioeconomic (8, 9), and geographic (10) disparities in rates of liver transplantation among patients with HCC. However, little is known about how patients navigate the early parts of the process, including referral and evaluation, in part because data prior to listing are not systematically reported (11). Understanding the steps at which patients experience barriers to care is critical to informing effective interventions to improve equity in access to transplant for patients with HCC.

In this study, we linked data on a subset of patients with HCC who either met the Milan criteria or downstaging criteria in Georgia from the Georgia Cancer Registry (GCR) to transplant center data from the only two liver transplant centers in the state. We describe rates of transplant referral, evaluation initiation, and evaluation completion in this population for the first time, and identify demographic factors associated with completion of each step.

## Materials and Methods

### Study Population and Data Sources

Data on incident HCC diagnosed in the state of Georgia were obtained from the GCR, part of a coordinated system of cancer registries funded by the NCI (Surveillance, Epidemiology, and End Results; SEER registries). SEER registries are noted for their data quality and completeness; prior studies have estimated over 97% completeness of hospital cancer case reporting (12). Data on transplant referral, evaluation, and listing were obtained from the two liver transplant centers in the state of Georgia: Emory Transplant Center and Piedmont Transplant Institute. Probabilistic linkage of these data sources was conducted by the GCR. This study was reviewed and approved by the Emory Institutional Review Board.

Our study population included incident, adult (18 ≤ age ≤ 75) patients with HCC in Georgia diagnosed between January 1, 2010 and December 31, 2019 and followed for outcomes (transplant, death, loss to follow-up) through December 31, 2020. Patients were excluded if they had a diagnosis date that occurred after they initiated evaluation with a transplant center (*n* = 184) or occurred more than 1 month after liver transplant referral (*n* = 32).

**FIGURE 1:**
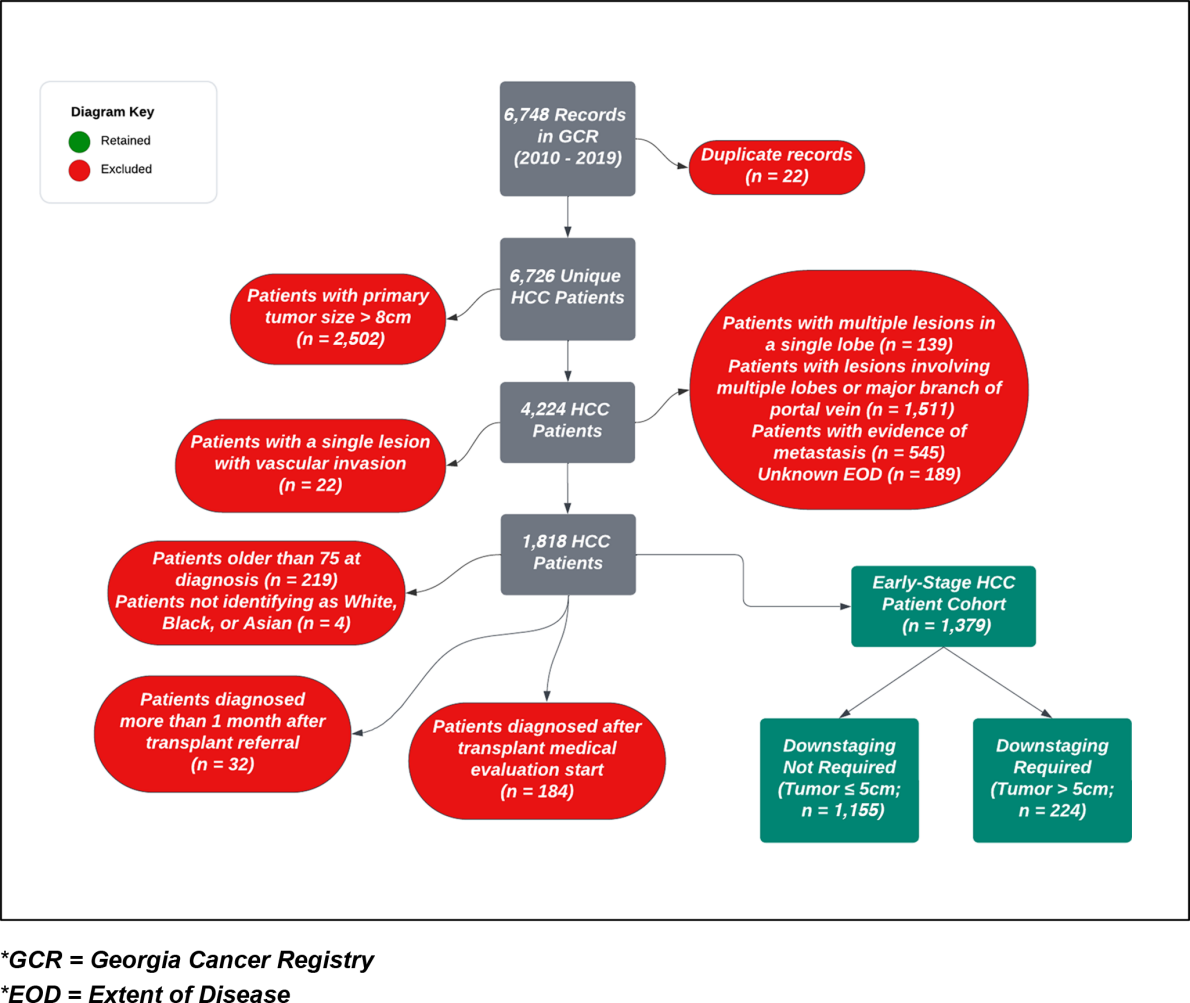
Defining the early-stage HCC cohort—inclusion and exclusion of patients diagnosed in Georgia, 2010–2019.

Cancer registry data formatting precluded full use of the most common liver transplant criteria, the Milan criteria, as inclusion parameters for our cohort. Cases were coded using the clinical tumor—node—metastasis (TNM) staging system. For inclusion, patients must have had no spread to lymph nodes (N0) or metastasis (M0). For T staging, single tumors were coded as T1, while cases coded as T2 could have either multiple tumors with none greater than 5 cm or a single tumor with vascular involvement. Because T2 patients may not fall within Milan criteria, we decided to only include T1 cases in our main analysis (Fig. 1). We conducted a sensitivity analysis including T2 patients with multiple tumors. Our size limit for T1 patients was set at ≤8 cm in accordance with downstaging protocols (13). Patients with tumors that were >5 but ≤8 cm were classified as “downstaging required for transplant.”

### Outcomes

Primary outcomes for this analysis were completion of each step of the transplant process prior to waitlisting: referral, evaluation initiation, and evaluation completion. Time to event was calculated as the time between the event date and the date of the prior event in the process (for referral, this was diagnosis). For patients referred to both transplant centers in our study, only the first referral following HCC diagnosis was included.

For 27 patients whose recorded diagnosis date occurred after referral but within 30 days of referral and prior to initiating transplant evaluation, referral was assumed to occur contemporaneously with diagnosis. These patients were retained in the analysis and assigned a referral time of 1 day. We conducted sensitivity analyses setting referral time to the midpoint value between referral and diagnosis and the absolute value between referral and diagnosis and determined our findings were robust to this decision.

### Exposures

Demographic factors hypothesized to be associated with access to transplant included age at diagnosis, gender, patient race (White, Black, or Asian), patient ethnicity (non-Hispanic or Hispanic), urbanicity (living in an urban commuting area or a non-urban commuting area), proportion of the census tract living in poverty (<5%, 5%–<10%, 10%–<20%, and 20%–100%), and insurance at cancer diagnosis [private, Medicaid (including patients dually enrolled in Medicare), Medicare, military insurance, uninsured, and unknown; the last three categories were collapsed for analyses]. Requirements for tumor downstaging were also hypothesized to be associated with transplant access.

Demographic variables were obtained from the GCR at the time of HCC diagnosis and include both patient- and provider-reported variables. Geographic characteristics (census tract poverty, urbanicity) were assigned by the GCR from patient addresses at the time of diagnosis.

### Statistical Analyses

We described demographic characteristics of our patient cohort and evaluated bivariate associations using *χ*^2^ tests, Fisher exact tests, or *t* tests as appropriate. To evaluate multivariable associations between demographic factors and completion of the selection process, we estimated the time to each event accounting for death as a competing risk using a cause-specific hazard model.

In sensitivity analyses, we reassessed multivariable associations between patient-level factors and step completion accounting for non-transplant cancer treatment (e.g., surgical resection, local tumor destruction) in addition to death as competing risks. In the first iteration of these analyses, patients with a history of resection or local tumor destruction were censored prior to transplant referral. Event dates for these treatments were not collected by transplant centers or the cancer registry; therefore, these patients were assigned the median referral time as their time to censorship. Among referred patients who received a non-transplant curative treatment, these patients were censored prior to evaluation initiation and assigned the median time to evaluation start as their time to censorship.

In a final sensitivity analysis, we described characteristics of patients who were reported as receiving a transplant to the GCR but who were not referred to one of our two transplant centers to evaluate potential selection bias from patients leaving the state to receive care. All analyses were conducted in R.

### Data Availability

The data analyzed in this study are available from GCR. Restrictions apply to the availability of these data, which were used under license for this study. Data are available upon reasonable request with the permission of the Georgia Department of Public Health.

## Results

### Study Cohort Characteristics

We identified 1,379 patients that met our primary cohort inclusion criteria. Approximately 75% of patients were male, with an average age at diagnosis of 62.3 years (Table 1). The most common insurance type was Medicare (46%), followed by private insurance (26%). Nearly two-thirds of patients (64%) were White, 31% were Black, and 6% were Asian.

**TABLE 1:** Distribution of demographic and clinical characteristics among patients with early-stage HCC diagnosed in Georgia (2010–2019)

	Early-stage cohort (*n* = 1,379)
Patient sex	
Female	350 (25.4%)
Male	1,029 (74.6%)
Age at diagnosis	
Mean (SD)	62.3 (8.2)
Tumor downstaging	
Not required	1,155 (83.8%)
Required	224 (16.2%)
Race	
White	877 (63.6%)
Black	426 (30.9%)
Asian	76 (5.5%)
Ethnicity	
Hispanic	60 (4.4%)
Non-Hispanic	1,319 (95.6%)
Urbanicity	
Non-UCA	186 (13.5%)
UCA	1,193 (86.5%)
Census tract poverty	
<5% Poverty	125 (9.1%)
<10% Poverty	228 (16.5%)
<20% Poverty	508 (36.8%)
≥20% Poverty	518 (37.6%)
Health insurance	
Private insurance	361 (26.2%)
Medicaid	169 (12.3%)
Medicare	628 (45.5%)
Other	221 (16.0%)

Abbreviation: UCA, urban commuting area.

**FIGURE 2:**
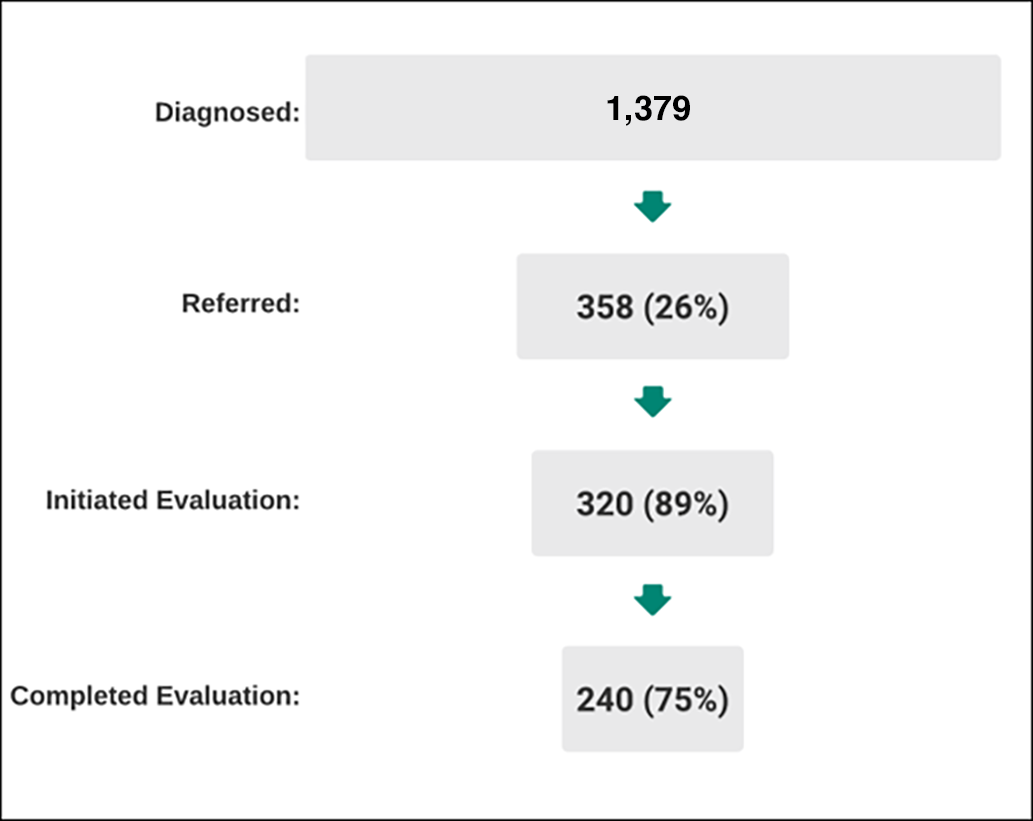
Liver transplant referral and evaluation among patients diagnosed with early-stage HCC in Georgia, 2010–2019.

Among included patients, 358 (26%) were ever referred to one of the two liver transplant centers in Georgia (Fig. 2). Of those referred, 89% (*n* = 320) initiated the evaluation and 75% of patients who initiated the evaluation completed the process (*n* = 240).

### Transplant Referral

Table 2 provides bivariate associations between patient demographic characteristics and liver transplant referral. Age (*P* < 0.001), insurance type (*P* < 0.001), and requirements for tumor downstaging (*P* < 0.001) were associated with referral status. Specifically, referred patients were younger at diagnosis compared with patients not referred (mean age: 60.4 vs. 63.0 years). Referred patients were also more likely to have private insurance (37%) compared with patients who were not referred for transplant (22%). Patients who required downstaging made up 8% of those referred for transplant compared with 19% of those not referred.

**TABLE 2:** Associations between early-stage HCC patient characteristics and liver transplant referral in Georgia (*n* = 1,379)

	Transplant referral status		
	Referred (*n* = 358)	Not Referred (*n* = 1,021)	*P*-value
Patient sex			
Male	258 (72.1%)	771 (75.5%)	0.22
Age at diagnosis			
Mean (SD)	60.4 (7.2)	63.0 (8.4)	<0.001
Tumor downstaging			
Not required	330 (92.2%)	825 (80.8%)	<0.001
Required	28 (7.8%)	196 (19.2%)	
Race			
White	231 (64.5%)	646 (63.3%)	0.80
Black	106 (29.6%)	320 (31.3%)	
Asian	21 (5.9%)	55 (5.4%)	
Ethnicity			
Hispanic	19 (5.3%)	41 (4.0%)	0.38
Non-Hispanic	339 (94.7%)	980 (96.0%)	
Urbanicity			
Non-UCA	44 (12.3%)	142 (13.9%)	0.50
UCA	314 (87.7%)	879 (86.1%)	
Census tract poverty			
<5% Poverty	41 (11.5%)	84 (8.2%)	0.05
<10% Poverty	70 (19.6%)	158 (15.5%)	
<20% Poverty	126 (35.2%)	382 (37.4%)	
≥20% Poverty	121 (33.8%)	397 (38.9%)	
Health insurance			
Private insurance	134 (37.4%)	227 (22.2%)	<0.001
Medicaid	40 (11.2%)	129 (12.6%)	
Medicare	148 (41.3%)	480 (47.0%)	
Other	36 (10.1%)	185 (18.1%)	

Abbreviation: UCA, urban commuting area.

In a multivariable model (Table 4), patients with Medicaid [cause-specific hazard ratio (csHR): 0.60, 95% confidence interval (CI): 0.41–0.86], Medicare (csHR: 0.72, 95% CI: 0.56–0.93), and other insurance (csHR: 0.42, 95% CI: 0.29–0.61) had a significantly lower cause-specific hazard of referral to transplant compared with patients with private insurance. Increasing age was also associated with lower cause-specific hazard of referral (csHR per year: 0.97, 95% CI: 0.96–0.98), as was requiring downstaging prior to transplant (csHR: 0.42, 95% CI: 0.29–0.62).

### Evaluation Initiation

Table 3 provides bivariate associations between demographic characteristics and the transplant evaluation process. Age at diagnosis (*P* = 0.03), patient race (*P* = 0.002), and census tract poverty (*P* = 0.002) were associated with evaluation initiation. Among referred patients, those who initiated the evaluation were younger compared with those who did not (mean age: 60.0 vs. 63.0 years). Black patients made up 27% of evaluation initiators compared with 55% of non-initiators, and 30% of patients who initiated the evaluation lived in census tracts with ≥20% of residents in poverty, compared with 63% of those who did not initiate the evaluation.

**TABLE 3:** Associations between early-stage HCC patient characteristics and liver transplant evaluation in Georgia

	Liver transplant evaluation step					
	Evaluation initiation (*n* = 358)			Evaluation completion (*n* = 320)		
	Initiated (*n* = 320)	Not Initiated (*n* = 38)	*P*-value	Completed (*n* = 240)	Not Completed (*n* = 80)	*P*-value
Patient sex						
Male	233 (72.8%)	25 (65.8%)	0.47	174 (72.5%)	59 (73.8%)	0.94
Age at diagnosis						
Mean (SD)	60.0 (7.1)	63.0 (7.5)	0.03	60.0 (7.3)	60.1 (6.4)	0.99
Tumor downstaging						
Not required	297 (92.8%)	33 (86.8%)	0.33	226 (94.2%)	71 (88.8%)	0.17
Required	23 (7.2%)	5 (13.2%)		14 (5.8%)	9 (11.3%)	
Race						
White	215 (67.2%)	16 (42.1%)	0.002	161 (67.1%)	54 (67.5%)	0.91
Black	85 (26.6%)	21 (55.3%)		63 (26.3%)	22 (27.5%)	
Asian	20 (6.3%)	1 (2.6%)		16 (6.7%)	4 (5.0%)	
Ethnicity						
Hispanic	18 (5.6%)	1 (2.6%)	0.69	14 (5.8%)	12 (5.0%)	1
Non-Hispanic	302 (94.4%)	37 (97.4%)		226 (94.2%)	76 (95.0%)	
Urbanicity						
Non-UCA	40 (12.5%)	4 (10.5%)	0.93	28 (11.7%)	12 (15.0%)	0.56
UCA	280 (87.5%)	34 (89.5%)		212 (88.3%)	68 (85.0%)	
Census tract poverty						
<5% Poverty	38 (11.9%)	3 (7.9%)	0.002	29 (12.1%)	9 (11.3%)	0.32
<10% Poverty	66 (20.6%)	4 (10.5%)		52 (21.7%)	14 (17.5%)	
<20% Poverty	119 (37.2%)	7 (18.4%)		93 (38.8%)	26 (32.5%)	
≥20% Poverty	97 (30.3%)	24 (63.2%)		66 (27.5%)	31 (38.8%)	
Health insurance						
Private insurance	124 (38.8%)	10 (26.3%)	0.26	99 (41.3%)	25 (31.3%)	0.04
Medicaid	33 (10.3%)	7 (18.4%)		18 (7.5%)	15 (18.8%)	
Medicare	130 (40.6%)	18 (47.9%)		98 (40.8%)	32 (40.0%)	
Other	33 (10.3%)	3 (7.9%)		25 (10.4%)	8 (10.0%)	

Abbreviation: UCA, urban commuting area.

Among those referred to transplant, Black patients had a 24% lower cause-specific hazard of initiating the evaluation (csHR: 0.76, 95% CI: 0.58–1.00) than White patients (Table 4). Patients living in census tracts with ≥20% of residents in poverty had a 38% lower cause-specific hazard of evaluation initiation compared with patients living in census tracts with <5% of residents in poverty (csHR: 0.62, 95% CI: 0.42–0.94). Increasing age was also associated with lower cause-specific hazard of evaluation initiation (csHR per year: 0.98, 95% CI: 0.97–1.00).

**TABLE 4:** Multivariable adjusted probability of transplant referral, evaluation initiation, and evaluation completion among patients with early-stage HCC in Georgia, accounting for death as a competing risk

	Transplant referral csHR (95% CI)	Evaluation initiation csHR (95% CI)	Evaluation completion csHR (95% CI)
Patient sex			
Female	Ref	Ref	Ref
Male	0.89 (0.70–1.12)	1.07 (0.83–1.37)	0.88 (0.66–1.17)
Age at diagnosis			
Unit = 1 year	0.97 (0.96–0.98)	0.98 (0.97–1.00)	0.99 (0.97–1.01)
Tumor downstaging			
Not required	Ref	Ref	Ref
Required	0.42 (0.29–0.62)	0.93 (0.60–1.43)	0.57 (0.33–1.00)
Race			
White	Ref	Ref	Ref
Black	1.03 (0.80–1.31)	0.76 (0.58–1.00)	1.10 (0.80–1.52)
Asian	0.83 (0.53–1.31)	1.11 (0.69–1.79)	1.19 (0.69–2.03)
Ethnicity			
Hispanic	Ref	Ref	Ref
Non-Hispanic	0.73 (0.45–1.17)	0.85 (0.51–1.40)	1.10 (0.63–1.93)
Urbanicity			
Non-UCA	Ref	Ref	Ref
UCA	0.96 (0.69–1.33)	1.03 (0.73–1.47)	1.05 (0.69–1.59)
Census tract poverty			
<5% Poverty	Ref	Ref	Ref
<10% Poverty	0.94 (0.64–1.39)	0.87 (0.57–1.32)	1.12 (0.70–1.78)
<20% Poverty	0.75 (0.53–1.08)	0.92 (0.63–1.34)	0.99 (0.64–1.52)
≥20% Poverty	0.72 (0.49–1.04)	0.62 (0.42–0.94)	0.80 (0.50–1.30)
Health insurance			
Private insurance	Ref	Ref	Ref
Medicaid	0.60 (0.41–0.86)	0.77 (0.51–1.16)	0.53 (0.32–0.89)
Medicare	0.72 (0.56–0.93)	1.17 (0.89–1.53)	1.06 (0.78–1.44)
Other	0.42 (0.29–0.61)	1.13 (0.76–1.69)	0.98 (0.62–1.54)

Abbreviation: UCA, urban commuting area.

### Evaluation Completion

Health insurance type was the only variable associated with completion of the transplant evaluation (Table 3). Among patients who completed the evaluation, 8% of patients had Medicaid, compared with 19% of those who did not complete the evaluation (*P* = 0.04). In multivariable analyses (Table 4), Medicaid patients had a nearly 50% lower cause-specific hazard of evaluation completion compared with patients with private insurance (csHR: 0.53, 95% CI: 0.32–0.89).

### Results Including T2 Patients

In our expanded cohort, we identified 1,471 adult patients diagnosed with HCC in Georgia between 2010 and 2019. Demographic characteristics of the additional 92 T2 patients in this cohort are described in the Supplementary Table S1. On average, T2 patients were older at HCC diagnosis compared with T1 patients (mean age: 64.0 vs. 62.3 years) and were more likely to require tumor downstaging to be eligible for transplant (100% vs. 16.2%). Rates of transplant referral (26%), evaluation initiation (89%), and evaluation completion (76%) were virtually unchanged in the expanded cohort (Supplementary Fig. S1).

Supplementary Table S2 provides multivariable associations between demographic factors and completion of each step of the transplant process among patients in the expanded cohort. Similar to the primary cohort, increasing age at diagnosis, requiring tumor downstaging, and non-private health insurance were associated with decreased cause-specific hazard of transplant referral. Notably, patients diagnosed with multiple tumors had a cause-specific hazard of transplant referral three times higher than patients diagnosed with a single tumor (csHR: 3.12, 95% CI: 1.84–5.27).

Also similar to the primary cohort, race and age were associated with cause-specific hazard of evaluation initiation. Living in a census tract with ≥20% of residents in poverty was associated with a lower cause-specific hazard of evaluation initiation compared with living in a census tract with <5% of residents in poverty but the association was not statistically significant in this cohort (csHR: 0.67, 95% CI: 0.45–1.01). Among those who initiated with evaluation, patients requiring tumor downstaging for transplant eligibility had 44% lower cause-specific hazard of evaluation completion compared with those not requiring downstaging (csHR: 0.56, 95% CI: 0.32–0.98).

### Sensitivity Analyses

In multivariable models accounting for non-transplant treatment and death as competing risks, findings were consistent with those from our primary analyses. In models where patients with history of resection, local tumor destruction, or other treatments (*n* = 375) were censored prior to transplant referral, increasing age (csHR: 0.97, 95% CI: 0.96–0.98), required downstaging (csHR: 0.41, 95% CI: 0.27–0.63), and non-private health insurance [Medicaid (csHR: 0.56, 95% CI: 0.37–0.83), Medicare (csHR: 0.65, 95% CI: 0.49–0.86), other (csHR: 0.43, 95% CI: 0.29–0.63)] were still associated with lower cause-specific hazard of transplant referral (Table 5).

**TABLE 5:** Multivariable adjusted probability of transplant referral, evaluation initiation, and evaluation completion among patients with early-stage HCC in Georgia, accounting for non-transplant cancer treatment and death as competing risks

	Patients Censored Before Transplant Referral			Patients Censored Following Transplant Referral		
	Transplant Referral csHR (95% CI)	Evaluation Initiation csHR (95% CI)	Evaluation Completion csHR (95% CI)	Transplant Referral csHR (95% CI)	Evaluation Initiation csHR (95% CI)	Evaluation Completion csHR (95% CI)
Patient sex						
Female	Ref	Ref	Ref	Ref	Ref	Ref
Male	0.80 (0.62–1.03)	0.94 (0.71–1.23)	0.78 (0.57–1.07)	0.89 (0.70–1.12)	0.90 (0.68–1.18)	0.78 (0.57–1.07)
Age at diagnosis						
Unit = 1 Year	0.97 (0.96–0.98)	0.99 (0.97–1.01)	0.99 (0.97–1.01)	0.97 (0.96–0.98)	0.98 (0.96–1.00)	0.99 (0.97–1.01)
Tumor downstaging						
Not required	Ref	Ref	Ref	Ref	Ref	Ref
Required	0.41 (0.27–0.63)	0.95 (0.59–1.54)	0.85 (0.49–1.50)	0.42 (0.29–0.62)	0.94 (0.58–1.52)	0.85 (0.49–1.50)
Race						
White	Ref	Ref	Ref	Ref	Ref	Ref
Black	0.95 (0.73–1.25)	0.73 (0.54–1.00)	1.08 (0.75–1.55)	1.03 (0.80–1.31)	0.71 (0.52–0.96)	1.08 (0.75–1.55)
Asian	0.78 (0.46–1.31)	0.93 (0.54–1.62)	1.41 (0.77–2.56)	0.83 (0.53–1.31)	0.82 (0.48–1.42)	1.41 (0.77–2.56)
Ethnicity						
Hispanic	Ref	Ref	Ref	Ref	Ref	Ref
Non-Hispanic	0.68 (0.40–1.15)	0.79 (0.45–1.38)	1.03 (0.57–1.86)	0.73 (0.45–1.17)	0.83 (0.48–1.45)	1.03 (0.57–1.86)
Urbanicity						
Non-UCA	Ref	Ref	Ref	Ref	Ref	Ref
UCA	0.96 (0.67–1.38)	1.00 (0.68–1.46)	1.04 (0.66–1.64)	0.96 (0.69–1.33)	0.99 (0.67–1.44)	1.04 (0.66–1.64)
Census tract poverty						
<5% Poverty	Ref	Ref	Ref	Ref	Ref	Ref
<10% Poverty	0.86 (0.56–1.32)	0.75 (0.47–1.19)	1.11 (0.66–1.86)	0.94 (0.64–1.39)	0.70 (0.44–1.12)	1.11 (0.66–1.86)
<20% Poverty	0.72 (0.49–1.06)	0.90 (0.59–1.36)	0.90 (0.56–1.43)	0.75 (0.53–1.08)	0.85 (0.56–1.29)	0.90 (0.56–1.43)
≥20% Poverty	0.70 (0.46–1.05)	0.58 (0.37–0.91)	0.79 (0.47–1.34)	0.72 (0.49–1.04)	0.57 (0.36–0.89)	0.79 (0.47–1.34)
Health insurance						
Private insurance	Ref	Ref	Ref	Ref	Ref	Ref
Medicaid	0.56 (0.37–0.83)	0.80 (0.51–1.25)	0.41 (0.23–0.74)	0.60 (0.41–0.86)	0.77 (0.49–1.20)	0.41 (0.23–0.74)
Medicare	0.65 (0.49–0.86)	1.21 (0.90–1.63)	0.98 (0.70–1.37)	0.72 (0.56–0.93)	1.13 (0.84–1.52)	0.98 (0.70–1.37)
Other	0.43 (0.29–0.63)	1.11 (0.73–1.68)	0.90 (0.56–1.43)	0.42 (0.29–0.61)	1.16 (0.76–1.76)	0.90 (0.56–1.43)

Abbreviation: UCA, urban commuting area.

In models where patients with history of non-transplant cancer treatments were censored following transplant referral (*n* = 63), cause-specific hazard estimates of referral were unchanged from primary analysis (Table 5). Similar to primary analyses, Black patients had a 29% lower cause-specific hazard of evaluation initiation compared with White patients (csHR: 0.71, 95% CI: 0.52–0.96), and patients living in census tracts with ≥20% of residents in poverty had a 43% lower cause-specific hazard of initiating the evaluation compared with patients living in census tracts with <5% of residents in poverty (csHR: 0.57, 95% CI: 0.36–0.89).

In our final sensitivity analysis (Table 6), we identified 45 patients recorded as receiving a transplant in the GCR but not referred to one of our two transplant centers. We compare demographic characteristics of these 45 patients with those who received a transplant in the state of Georgia (*n* = 146). Black patients made up 25% of those who received a transplant in Georgia compared with 16% outside the state. Patients living in census tracts with ≥20% poverty made up 30% of those who received a transplant in Georgia compared with 20% of those outside the state, and patients with Medicaid made up 6% of those who received a transplant in Georgia compared with 11% of those receiving one outside the state. None of these differences were statistically significant.

**TABLE 6:** Characteristics of patients with early-stage HCC transplanted at a Georgia transplant center versus those transplanted outside of Georgia (*n* = 191)

	Georgia transplant (*n* = 146)	Out of state transplant (*n* = 45)	*P*-value
Patient sex			
Male	108 (74.0%)	34 (75.6%)	0.99
Age at diagnosis			
Mean (SD)	59.4 (8.0)	60.4 (7.7)	0.44
Tumor downstaging			
Not required	137 (93.8%)	44 (97.8%)	0.51
Required	9 (6.2%)	1 (2.2%)	
Race			
White	100 (68.5%)	35 (77.8%)	0.48
Black	36 (24.7%)	7 (15.6%)	
Asian	10 (6.8%)	3 (6.7%)	
Ethnicity			
Hispanic	10 (6.8%)	2 (4.4%)	0.82
Non-Hispanic	136 (93.2%)	43 (95.6%)	
Urbanicity			
Non-UCA	16 (11.0%)	6 (13.3%)	0.87
UCA	130 (89.0%)	39 (86.7%)	
Census tract poverty			
<5% Poverty	18 (12.3%)	6 (13.3%)	0.23
<10% Poverty	31 (21.2%)	6 (13.3%)	
<20% Poverty	54 (37.0%)	24 (53.3%)	
≥20% Poverty	43 (29.5%)	9 (20.0%)	
Health insurance			
Private insurance	72 (49.3%)	16 (35.6%)	0.18
Medicaid	9 (6.2%)	5 (11.1%)	
Medicare	51 (34.9%)	16 (35.6%)	
Other	14 (9.6%)	8 (17.8%)	

Abbreviation: UCA, urban commuting area.

## Discussion

Among patients diagnosed with a single HCC tumor that was either within Milan or downstaging criteria in the state of Georgia, 26% were referred to one of the two liver transplant centers in the state. Age, insurance type, and downstaging requirements were associated with referral to transplant, race, and census tract poverty were associated with evaluation initiation, and Medicaid was associated with evaluation completion. Our findings highlight opportunities to improve access to transplant among T1 patients with HCC in Georgia, including improving referring provider knowledge about transplant insurance requirements, identifying barriers to evaluation initiation among Black patients and patients living in high poverty neighborhoods, and streamlining evaluation completion for Medicaid patients.

The optimal proportion of patients with HCC that should be referred to transplant is unknown. In our study, we found that 26% of patients diagnosed with a single HCC tumor that was either within Milan or downstaging criteria were referred to transplant, and an additional 23% received another form of curative therapy (i.e., resection or local tumor destruction), leaving 51% of the cohort with no curative treatment. Some of the patients who did not receive either form of therapy may have had an absolute contraindication to transplant, such as severe cardiac disease (3). However, most transplant criteria, including length of sobriety and degree of social support, are subjective and vary across transplant programs. Encouraging referring providers to err on the side of referral may result in an increased burden on transplant center staff by increasing the number of patients requiring evaluation. However, promoting referral also ensures that patients are not inappropriately ruled out for transplant by their referring provider before having the opportunity to be considered by the transplant team.

To our knowledge, this is the first study of referral to liver transplant among patients with HCC, although two previous studies have evaluated referral to treatment. One study using cancer registry data linked to Medicare from 1998 to 2007 found that referral to a “specialist” (including hepatology, oncology, surgery, or interventional radiology) was less likely for Black patients and more likely for patients living in the Northeast (14). However, this study did not link to any transplant center data. The second study linking data from the Pennsylvania Cancer Registry to hospital claims from 2006 to 2011 found that for patients diagnosed with HCC, referral to surgery (including ablation, resection, and transplantation) was less likely for older patients and patients with Medicaid (10). Neither of these studies examined referral to transplant specifically.

Similar to the Pennsylvania study, we found that insurance type was associated with likelihood of transplant referral. This is consistent with the findings of a recent scoping review of inequities in transplant access (15). While both transplant centers in our study accept transplant candidates with Medicaid or Medicare, patients with these insurance types were markedly less likely to be referred to transplant than privately insured patients. Referring providers may not be aware of transplant insurance requirements and decline to refer patients that may otherwise be eligible for transplantation due to their insurance coverage.

Previous single-center studies have examined completion of evaluation steps among referred patients. Our study found a notably higher proportion of patients initiated the evaluation (91%) than a prior study in Indiana (57%; ref. 16) or a survey of patients in North Carolina (49%; ref. 17). This may be because we focused on patients with early-stage HCC, as opposed to all end-stage liver disease patients, who may be more able or willing to initiate the transplant process. While not statistically significant, we observed an association in our study between race, socioeconomic status, and evaluation initiation. This is in contrast to the Indiana study, which did not find any differences in the proportion of patients from vulnerable populations who progressed to evaluation initiation. Similarly, while the Indiana study observed disparities in listing rates among patients with Medicaid and living in high poverty neighborhoods, race was not associated with listing in their study (16). This discrepancy indicates the potential for variation in disparities across the country and across transplant programs, and highlights the need for additional studies in this area.

While cancer registry data are a powerful tool for population-based analyses, there are limitations to its use for clinical research. The primary focus of cancer registries is cancer surveillance, and some variables that are important for clinical hepatology research, such as comorbidities or degree of liver dysfunction, are not systematically collected. In addition, staging methodologies used by cancer registries such as TNM staging may not perfectly map to clinical staging methods such as the Milan criteria. Although there have been major improvements in probabilistic record linkage (18), our observed referral rates may be underreported if patients were unable to be linked between registry and transplant center data. We were also unable to estimate referral and evaluation for patients who sought transplant care outside of the state of Georgia. In a sensitivity analysis, we determined that patients who received a transplant outside of Georgia were less likely to live in high poverty census tracts. This analysis indicates that the magnitude of our findings on inequities may be underestimated, and is consistent with prior research demonstrating that patients with lower socioeconomic status are less likely to travel outside of their own region to pursue transplantation (19). National sharing of referral and evaluation data would facilitate these types of studies in the future to gain a full understanding of the landscape of transplant care (11).

In summary, we found that among patients with HCC with a single tumor meeting either Milan or downstaging criteria in Georgia, non-privately insured patients were significantly less likely to be referred for liver transplant than those with private insurance. Black patients and patients living in high poverty neighborhoods were less likely to initiate the evaluation, and Medicaid patients were less likely to complete the evaluation. Our findings emphasize unique barriers to liver transplant access at each step in the process and the need for targeted interventions to improve equity in access.

## Supplementary Material

Figure S1 Liver transplant referral and evaluation among Georgia HCC patients identified in the expanded cohort (2010 – 2019)

Table S1 Distribution of demographic and clinical characteristics among HCC patients diagnosed in Georgia (2010 – 2019)

Table S2 Multivariable adjusted probability of transplant referral, evaluation initiation and evaluation completion among HCC patients in Georgia accounting for death as a competing risk
