# Supplementary figures and images for: Disparities in Access to Liver Transplant Referral and Evaluation among Patients with Hepatocellular Carcinoma in Georgia
Source: Cancer Res Commun. 2024 Apr 22;4(4):1111–9. doi: 10.1158/2767-9764.CRC-23-0541 (PMC11034460; doi:10.1158/2767-9764.CRC-23-0541)

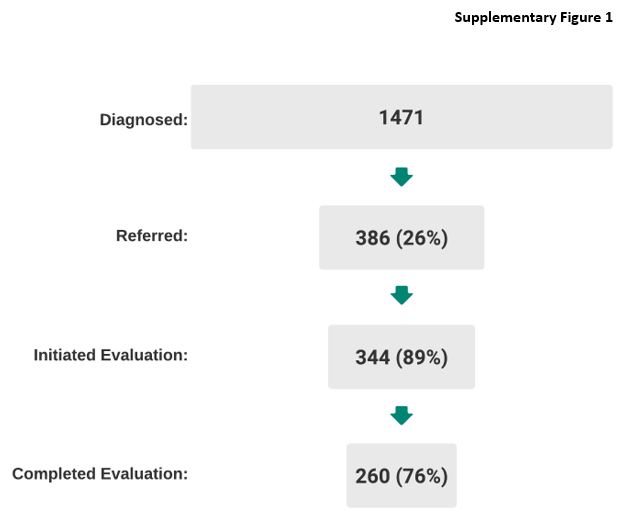

Supplement: Figure S1 — Liver transplant referral and evaluation among Georgia HCC patients identified in the expanded cohort (2010 – 2019) [file crc-23-0541-s01.png]
