# Supplementary material for: Disparities in Access to Liver Transplant Referral and Evaluation among Patients with Hepatocellular Carcinoma in Georgia
Source: Cancer Res Commun. 2024 Apr 22;4(4):1111–9. doi: 10.1158/2767-9764.CRC-23-0541 (PMC11034460; doi:10.1158/2767-9764.CRC-23-0541)
Supplement: Table S1 — Distribution of demographic and clinical characteristics among HCC patients diagnosed in Georgia (2010 – 2019) [file crc-23-0541-s02.docx]

**Supplementary Table 1. Distribution of demographic and clinical characteristics among HCC patients diagnosed in Georgia (2010 – 2019)**

|  | **Expanded HCC Cohort** | | |  |
| --- | --- | --- | --- | --- |
|  | **Single Tumor**  **(n = 1379)** | | **Multiple Tumors**  **(n = 92)** | **P-value** |
| ***Patient Sex*** | |  | | |
| Male | 1029 (74.6%) | | 70 (76.1%) | 0.85 |
| ***Age at Diagnosis*** | | | | |
| Mean (SD) | 62.3 (8.2) | | 64.0 (6.2) | 0.02 |
| ***Tumor Downstaging*** | | | | |
| Not Required | 1155 (83.8%) | | 0 (0%) | < 0.001 |
| Required | 224 (16.2%) | | 92 (100%) |  |
| ***Race*** | | | | |
| White | 877 (63.6%) | | 64 (69.6%) | 0.33 |
| Black | 426 (30.9%) | | 26 (28.3%) |  |
| Asian | 76 (5.5%) | | 2 (2.2%) |  |
| ***Ethnicity*** | | | | |
| Hispanic | 60 (4.4%) | | 3 (3.3%) | 0.82 |
| Non-Hispanic | 1319 (95.6%) | | 89 (96.7%) |  |
| ***Urbanicity*** | | | | |
| Non UCA | 186 (13.5%) | | 16 (17.4%) | 0.37 |
| UCA | 1193 (86.5%) | | 76 (82.6%) |  |
| ***Census Tract Poverty*** | | | | |
| < 5% Poverty | 125 (9.1%) | | 5 (5.4%) | 0.46 |
| < 10% Poverty | 228 (16.5%) | | 17 (18.5%) |  |
| < 20% Poverty | 508 (36.8%) | | 30 (32.6%) |  |
| ≥ 20% Poverty | 518 (37.6%) | | 40 (43.5%) |  |
| ***Health Insurance*** | | | | |
| Private Insurance | 361 (26.2%) | | 21 (22.8%) | 0.66 |
| Medicaid | 169 (12.3%) | | 10 (10.9%) |  |
| Medicare | 628 (45.5%) | | 42 (45.7%) |  |
| Other | 221 (16.0%) | | 19 (20.7%) |  |
