# Supplementary material for: Disparities in Access to Liver Transplant Referral and Evaluation among Patients with Hepatocellular Carcinoma in Georgia
Source: Cancer Res Commun. 2024 Apr 22;4(4):1111–9. doi: 10.1158/2767-9764.CRC-23-0541 (PMC11034460; doi:10.1158/2767-9764.CRC-23-0541)
Supplement: Table S2 — Multivariable adjusted probability of transplant referral, evaluation initiation and evaluation completion among HCC patients in Georgia accounting for death as a competing risk [file crc-23-0541-s03.docx]

**Supplementary Table 2. Multivariable adjusted probability of transplant referral, evaluation initiation and evaluation completion among HCC patients in Georgia accounting for death as a competing risk**

|  | **Transplant Referral**  **{csHR (95% CI)}** | **Evaluation Initiation**  **{csHR (95% CI)}** | **Evaluation Completion**  **{csHR (95% CI)}** |
| --- | --- | --- | --- |
| ***Patient Sex*** | | | |
| Female | *Ref* | *Ref* | *Ref* |
| Male | 0.89 (0.71, 1.12) | 1.08 (0.85, 1.38) | 0.85 (0.64, 1.13) |
| ***Age at Diagnosis*** | | | |
| Unit = 1 Year | 0.97 (0.96, 0.98) | 0.98 (0.97, 1.00) | 0.99 (0.97, 1.01) |
| ***Tumor Count*** | | | |
| Single | *Ref* | *Ref* | *Ref* |
| Multiple | 3.12 (1.84, 5.27) | 1.10 (0.61, 1.97) | 1.58 (0.78, 3.19) |
| ***Tumor Downstaging*** | | | |
| Not Required | *Ref* | *Ref* | *Ref* |
| Required | 0.42 (0.29, 0.63) | 0.93 (0.60, 1.42) | 0.56 (0.32, 0.98) |
| ***Race*** | | | |
| White | *Ref* | *Ref* | *Ref* |
| Black | 0.98 (0.77, 1.24) | 0.78 (0.60, 1.02) | 1.11 (0.81, 1.51) |
| Asian | 0.87 (0.56, 1.36) | 0.99 (0.62, 1.59) | 1.23 (0.73, 2.06) |
| ***Ethnicity*** | | | |
| Hispanic | *Ref* | *Ref* | *Ref* |
| Non-Hispanic | 0.74 (0.46, 1.18) | 0.85 (0.52, 1.38) | 1.08 (0.63, 1.86) |
| ***Urbanicity*** | | | |
| Non UCA | *Ref* | *Ref* | *Ref* |
| UCA | 1.00 (0.73, 1.37) | 1.06 (0.75, 1.49) | 1.04 (0.70, 1.55) |
| ***Census Tract Poverty*** | | | |
| < 5% Poverty | *Ref* | *Ref* | *Ref* |
| < 10% Poverty | 1.04 (0.71, 1.51) | 0.94 (0.63, 1.40) | 1.09 (0.69, 1.71) |
| < 20% Poverty | 0.81 (0.57, 1.15) | 0.90 (0.62, 1.31) | 0.94 (0.62. 1.43) |
| ≥ 20% Poverty | 0.72 (0.50, 1.04) | 0.67 (0.45, 1.01) | 0.79 (0.50, 1.27) |
| ***Health Insurance*** | | | |
| Private Insurance | *Ref* | *Ref* | *Ref* |
| Medicaid | 0.59 (0.42, 0.84) | 0.70 (0.47, 1.04) | 0.53 (0.32, 0.87) |
| Medicare | 0.70 (0.54, 0.89) | 1.15 (0.89, 1.49) | 1.03 (0.77, 1.39) |
| Other | 0.41 (0.28, 0.58) | 1.01 (0.69, 1.49) | 1.02 (0.66, 1.58) |
